# Neutrophil count and percentage: potential independent prognostic indicators for advanced cancer patients in a palliative care setting

**DOI:** 10.18632/oncotarget.16469

**Published:** 2017-03-22

**Authors:** Weiwei Zhao, Peng Wang, Huixun Jia, Menglei Chen, Xiaoli Gu, Minghui Liu, Zhe Zhang, Wenwu Cheng, Zhenyu Wu

**Affiliations:** ^1^ Department of Integrated Therapy, Fudan University Shanghai Cancer Center, Shanghai, China; ^2^ Department of Oncology, Shanghai Medical College, Fudan University, Shanghai, China; ^3^ Department of Integrative Oncology, Fudan University Shanghai Cancer Center, Shanghai, China; ^4^ Center for Biomedical Statistical, Fudan University Shanghai Cancer Center, Shanghai, China; ^5^ Department of Biostatistics, School of Public Health, Key Laboratory of Public Health Safety and Collaborative Innovation Center of Social Risks Governance in Health, Fudan University, Shanghai, China

**Keywords:** neutrophil count, neutrophil percentage, prognostic value, advanced cancer, palliative care

## Abstract

The purpose of this study was to evaluate the count and percentage of neutrophils as prognostic indicators in advanced cancer patients undergoing palliative care. 378 consecutive patients receiving treatment at the palliative care unit of Fudan University Shanghai Cancer Center between July 2013 and October 2015 were reviewed. In 106 of these patients, the data were extended during the follow-up. The cut-off values selected for the neutrophil count and percentage were 8.0×109/L and 85%, respectively. Both a high pretreatment neutrophil count (*HR* = 1.828, 95% CI: 1.409∼2.371, *P*<0.001) and a high pretreatment neutrophil percentage (*HR* = 1.475, 95% CI: 1.106∼1.967, *P=*0.008) were independent prognostic factors for decreased overall survival. Furthermore, in the follow-up cohort of readmitted patients (*n* = 106), patients with a newly increased neutrophil count or percentage were respectively, 1.837 (95% CI: 1.096∼3.079) and 3.268 (95% CI: 1.848∼5.778) times more likely to have a poor prognosis compared with patients with low neutrophil conditions (*P*=0.021, *P*<0.001). In conclusions, both high pretreatment or newly increased count and percentage of neutrophils were confirmed as independent prognostic factors for adverse outcomes. These parameters may be used as stratification factors in identifying advanced cancer patients with poor prognosis in palliative care settings.

## INTRODUCTION

Cancer has become a global epidemic that constitutes an enormous burden on society. According to data from GLOBOCAN 2012, approximately 14.1 million new cancer cases and 8.2 million deaths occurred, which accounted for an estimated 13% of all deaths worldwide [1]. Cancer can lead to severe health consequences, especially in patients with advanced cancer [2]. Therefore, early recognition and risk stratification are imperative to improve the outcomes in patients with advanced cancer. However, to the best of our knowledge, the ability to predict the prognosis of patients with advanced cancer is still poor. Over the past few decades, several prognostic models have been proposed, such as the Palliative Prognostic Score, Palliative Prognostic Index, Palliative Performance Scale, and the Glasgow Prognostic Score, which may be used to predict the survival of patients with advanced cancer [3]. Although these prognostic models are somewhat well-validated, they are too complicated in that they require a comprehensive disease assessment and hematologic assay to generalize their clinical application. Therefore, it is of great value to search for highly accurate and easily detectable indicators to predict the prognosis of patients with advanced cancer.

Increasing evidence suggests that inflammation plays a key role in the initiation and progression of cancer [4]. Biomarkers of inflammation, such as C-reactive protein (CRP), neutrophil count (NC), and neutrophil-to-lymphocyte ratio (NLR), have been studied as important tools for risk stratification in cancers associated with increased tumor burden and aggressive tumor biology [5–7]. In recent years, an elevated NC has been observed to be an independent predictor of poor clinical outcome in patients with diverse cancer types, most notably in gastric cancer, nasopharyngeal carcinoma, urinary tract carcinoma, lung and ovarian cancer [6, 8–10]. However, the relationship between the percentage of neutrophils and survival of cancers is rarely studied and only found in individuals with hepatocellular carcinoma or nasopharyngeal carcinoma [11, 12]. Besides, controversial issues were also found in prostate cancer patients that NC does not correlate with biochemical recurrence and disease free survival while neutrophil percentage (NP) does [13]. Moreover, no study to date has investigated the association between the count or percentage of peripheral neutrophils and the mortality of patients with advanced cancer.

Thus, the aim of this study was to examine and compare the impact of the pretreatment peripheral blood neutrophil count and percentage on overall survival (OS) of patients with advanced cancer in a palliative setting; and to promote the use of dynamic changes in NC and NP for risk stratification with a clinical practice of palliative care.

## RESULTS

### Patient characteristics

A total of 378 qualified patients were retrieved from the database. Of the 378 patients, 106 with readmission data were selected for cohort 2. The flow diagram of the two study cohorts were described in our previous study [14].

A general description of the patients in cohort 1 and cohort 2 is given in Table 1 and Table 2, respectively. The median survival time was 51 days (95% CI: 41∼61) days and 143 (95% CI: 107∼179) days, respectively. The median age of the patients in cohort 1 was 64 years (rang, 14 to 94 years). Males comprised the majority of the patients in both cohorts, and only a few patients had stage III (6.08%) disease. The most common primary tumors were gastrointestinal (52.38%), thoracic (22.75%) and urogenital (15.61%). Head & neck neoplasms (4.23%) and other tumors (5.03%) constituted a minority of tumor types. Approximately 30% of patients had a family history of cancer, 40% of patients had comorbidities, and 70% of patients exhibited poor nutritional status. Ninety-nine patients received palliative chemoradiotherapy (PCR) and best supportive care (BSC), while the remaining 279 patients received BSC only. Ninety-nine patients received palliative chemoradiotherapy (PCR), while the remaining 279 patients received best supportive care (BSC) only. The proportion of patients in good physical condition (ECOG < 3) in cohort 2 was clearly higher than in cohort 1. The median duration of follow-up for patients in cohort 1 and cohort 2 was 445 days (range, 1∼882 days) and 509 days (range, 28∼882 days), respectively.

**Table 1:** Comparisons of baseline clinicopathological features based on NC and NP in cohort 1 (*N*=378)

Clinicopathological features	*N* (%)	NC		*P* value	NP		*P* value
		LNC(*n*= 254)	HNC(*n*= 122)		LNP(*n*= 297)	HNP(*n*= 81)	
Age(Mean±SD)	378	63.78±12.74	63.07±12.97	0.6108	63.58±13.03	63.41±12.00	0.9133
Gender				0.1012			0.1886
Male	209(55.29%)	133(52.36%)	76(61.29%)		159(53.54%)	50(61.73%)	
Female	169(44.71%)	121(47.64%)	48(38.71%)		138(46.46%)	31(38.27%)	
Tumor stage				0.1042			0.0394
III	23(6.08%)	19(7.48%)	4(3.23%)		22(7.41%)	1(1.23%)	
IV	355(93.92%)	235(92.52%)	120(96.77%)		275(92.59%)	80(98.77%)	
Primary tumor site				0.5800			0.6654
Gastrointestinal tumors	198(52.38%)	131(51.57%)	67(54.03%)		152(51.18%)	46(56.79%)	
Thoracic cancers	86(22.75%)	55(21.65%)	31(25.00%)		71(23.91%)	15(18.52%)	
Urogenital neoplasms	59(15.61%)	45(17.72%)	14(11.29%)		48(16.16%)	11(13.58%)	
Head and neck neoplasm	16(4.23%)	11(4.33%)	5(4.03%)		11(3.70%)	5(6.17%)	
Other tumors	19(5.03%)	12(4.72%)	7(5.65%)		15(5.05%)	4(4.94%)	
Palliative care				0.7042			0.7292
PCR	279(73.81%)	189(74.41%)	90(72.58%)		218(73.40%)	61(75.31%)	
BSC	99(26.19%)	65(25.59%)	34(27.42%)		79(26.60%)	20(24.69%)	
Family history				0.6376			0.6156
No	264(70.78%)	181(71.54%)	83(69.17%)		207(70.17%)	57(73.08%)	
Yes	109(29.22%)	72(28.46%)	37(30.83%)		88(29.83%)	21(26.92%)	
Unknown	5						
ECOG score				0.0107			0.0005
<3	218(57.67%)	158(62.20%)	60(48.39%)		185(62.29%)	33(40.74%)	
>=3	160(42.33%)	96(37.80%)	64(51.61%)		112(37.71%)	48(59.26%)	
Comorbidity				0.9782			0.6208
No	229(60.58%)	154(60.63%)	75(60.48%)		178(59.93%)	51(62.96%)	
Yes	149(39.42%)	100(39.37%)	49(39.52%)		119(40.07%)	30(37.04%)	
Nutritional status				0.5897			0.0831
Normal	107(28.31%)	76(29.92%)	31(25.00%)		91(30.64%)	16(19.75%)	
Abnormal	268(70.90%)	176(69.29%)	92(74.19%)		204(68.69%)	64(79.01%)	
Unknown	3(0.79%)	2(0.79%)	1(0.81%)		2(0.67%)	1(1.23%)	

Abbreviation: NC, neutrophil counts; NP, neutrophil percentages; SD, Standard Deviation;

LNC, low NC (pretreatment NC<=8); HNC, high NC (pretreatment NC>8); LNP, low NP (pretreatment NP<=0.85); HNP, high NP (pretreatment NP>0.85); BSC, best supportive care; PCR, palliative chemoradiotherapy; ECOG, Eastern Cooperative Oncology Group

**Table 2:** Comparisons of baseline clinicopathological features based on changes in NC and NP in cohort 2 (N=106)

Clinicopathological features	*N* (%)	NC		*P* value	NP		*P* value
		Descending	Ascending		Descending	Ascending	
Age(Mean±SD)	106	61.82±11.28	63.94±11.72	0.3648	59.95±12.15	65.27±10.72	0.0198
Gender				0.8730			0.4712
Male	56(52.83%)	21(53.85%)	35(52.24%)		24(57.14%)	32(50.00%)	
Female	50(47.17%)	18(46.15%)	32(47.76%)		18(42.86%)	32(50.00%)	
Tumor stage				0.0096			0.0198
III	13(12.26%)	9(23.08%)	4(5.97%)		9(21.43%)	4(6.25%)	
IV	93(87.74%)	30(76.92%)	63(94.03%)		33(78.57%)	60(93.75%)	
Primary tumor site				0.1882			0.4884
Gastrointestinal tumors	60(56.60%)	21(53.85%)	39(58.21%)		24(57.14%)	36(56.25%)	
Thoracic cancers	14(13.21%)	5(12.82%)	9(13.43%)		4(9.52%)	10(15.63%)	
Urogenital neoplasms	23(21.70%)	11(28.21%)	12(17.91%)		12(28.57%)	11(17.19%)	
Head and neck neoplasm	6(5.66%)	0(0.00%)	6(8.96%)		1(2.38%)	5(7.81%)	
Other tumors	3(2.83%)	2(5.13%)	1(1.49%)		1(2.38%)	2(3.13%)	
Palliative care				<0.001			0.0025
PCR	52(49.06%)	9(23.08%)	43(64.18%)		13(30.95%)	39(60.93%)	
BSC	54(50.94%)	30(76.92%)	24(35.82%)		29(69.05%)	25(39.06%)	
Family history				0.1718			0.1872
No	73(68.87%)	30(76.92%)	43(64.18%)		32(76.19%)	41(64.06%)	
Yes	33(31.13%)	9(23.08%)	24(35.82%)		10(23.81%)	23(35.94%)	
ECOG score				0.1292			0.1741
<3	81(76.42%)	33(84.62%)	48(71.64%)		35(83.33%)	46(71.88%)	
>=3	25(23.58%)	6(15.38%)	19(28.36%)		7(16.67%)	18(28.13%)	
Comorbidity				0.4552			0.6747
No	63(59.43%)	25(64.10%)	38(56.72%)		26(61.90%)	37(57.81%)	
Yes	43(40.57%)	14(35.90%)	29(43.28%)		16(38.10%)	27(42.19%)	
Nutritional status				0.2475			0.6494
Normal	42(39.62%)	19(48.72%)	23(34.33%)		19(45.24%)	23(35.94%)	
Abnormal	63(59.43%)	20(51.28%)	43(64.18%)		23(54.76%)	40(62.50%)	
Unknown	1(0.94%)	0(0.00%)	1(1.49%)		0(0.00%)	1(1.56%)	

Abbreviation: NC, neutrophil counts; NP, neutrophil percentages; SD, Standard Deviation; BSC, best supportive care; PCR, palliative chemoradiotherapy; ECOG, Eastern Cooperative Oncology Group.

### Determination of the cut-off value of NC and NP

**Figure 1:**
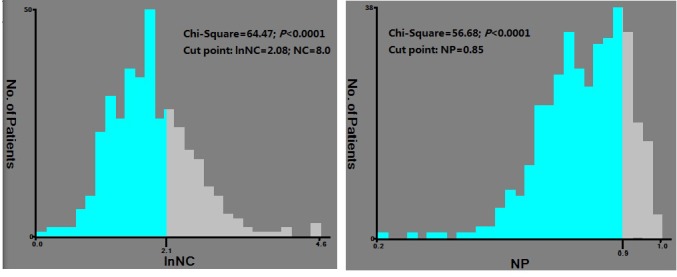
X-tile analysis was performed to determine the optimal cut-off values using the data of cohort 1 The optimal cut-off value for NC and NP were 8.0 (x2 = 64.47, p < 0.0001) and 0.85 (x2 = 58.68, p < 0.0001), respectively.

The conversion of a continuous variable into a binary one is common in clinical settings. Patients are simply classified into “High” and “Low” groups, which may be convenient for diagnosis or for the prediction of prognosis. However, acknowledged clinical cut-off values were not available for NC or NP in patients with advanced cancer. Based on a minimal p-value algorithm using X-tile [15], the optimal cut-off values for NC and NP were 8.0*109/L and 85%, respectively (Figure 1). Accordingly, patients with a pretreatment NC greater than 8 were categorized into the “high NC” (HNC) group, patients with a pretreatment NC less than or equal to 8 were categorized into the “low NC” (LNC) group. Patients with a pretreatment NP greater than 0.85 were categorized into the “high NP” (HNP) group, while patients with a pretreatment NP less than or equal to 0.85 were categorized into the “low NP” (LNP) group. The C-index was 0.73 (95%CI 0.71∼0.76) in the NC model and 0.72 (95%CI 0.70∼0.75) in the NP model, respectively.

### Association of NC, NP and the changes in these values with clinicopathological features

The associations between clinicopathological features and the values (low *vs*. high) of NC and NP from cohort 1 are listed in Table 1. Most of the enrolled patients had a low NC (67.20%) and a low NP (78.57%). Generally, proportions of patients with certain clinicopathological features in the LNC and HNC group were similar to the proportions in the LNP and HNP groups, and no differences were observed with respect to age, gender, tumor site, family history, palliative care, nutritional status or the presence of comorbidities. However, the ECOG score was the only clinicopathological feature that was significantly associated with both NC and NP. Obviously, patients with low NC or NP had a better physical status (ECOG < 3). In cohort 2, we first calculated the changes in NC and NP, and then, the patients were categorized into either the descending group (change < 0) or the ascending group (change > 0). Tumor stage and palliative care were the only features that were significant in the ascending and descending NC and NP groups. Most patients with stage III disease or BSC exhibited a decreased NC and NP, while most patients with stage IV disease or PCR showed an increased NC and NP. No other statistically significant differences in proportions of patients with specific clinicopathological features were observed between the descending and ascending groups (Table 2).

### Association of NC, NP and the changes in these values with OS

**Figure 2:**
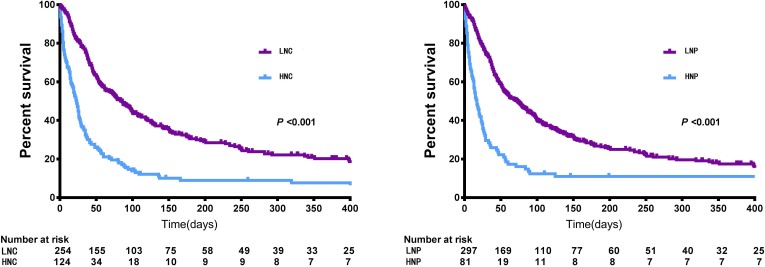
Overall survival of patients under palliative care stratified by pretreatment NC and NP (cohort 1) Abbreviation: NC, neutrophil counts; NP, neutrophil percentages; LNC, low NC (pretreatment NC < = 8); HNC, high NC (pretreatment NC > 8); LNP, low NP (pretreatment NP < = 0.85); HNP, high NP (pretreatment NP > 0.85).

Patients in cohort 1 were divided into the HNC or LNC groups and the HNP or LNP groups. According to the Kaplan-Meier analysis, the LNC and LNP groups experienced a significantly prolonged survival (median survival time: 83 (95% CI: 62-100) days *vs*. 23 (16-27) days, *P* < 0.001; and 72 (53-87) days *vs*. 17 (12-24) days, *P* < 0.001) (Figure 2). Multivariable Cox proportional hazard models demonstrated that primary gender, tumor stage, albumin, LDH, ECOG score and NC / NP were independent prognostic factors of OS. After controlling for important confounding variables, NC (HNC *vs*. LNC, *HR*: 1.828, 95% CI: 1.409∼2.371) / NP (HNP *vs*. LNP, *HR*: 1.475, 95% CI: 1.106∼1.967) remained significantly associated with OS (Table 3).

**Table 3:** Multivariate Cox regression analysis for NC and NP (*N*=378)

Prognostic factors	NC Model*		NP Model*	
	Adjusted HR (95%CI)	*P* value	Adjusted HR (95%CI)	*P* value
Gender (female vs. male)	1.358(1.069∼1.725)	0.012	1.356(1.068∼1.723)	0.012
Age	1.002(0.992∼1.012)	0.711	0.998 (0.989∼1.008)	0.764
Primary tumor site	0.915(0.821∼1.019)	0.105	0.905(0.812∼1.007)	0.068
Tumor stage (IV vs. III)	4.410(2.037∼9.543)	<0.001	4.303(1.990∼9.303)	<0.001
Family history (Yes vs. No)	0.994(0.768∼1.287)	0.967	1.026(0.793∼1.327)	0.846
Nutrient (Abnormal vs. Normal)	1.219(0.948∼1.567)	0.122	1.183(0.919∼1.525)	0.192
ECOG	1.658(1.294∼2.124)	<0.001	1.621(1.264∼2.078)	<0.001
Comorbidity (Yes vs. No)	0.860(0.663∼1.116)	0.256	0.893(0.689∼1.158)	0.394
Palliative care(BSC vs PCR)	1.012(0.778∼1.315)	0.930	1.033(0.795∼1.342)	0.808
Albumin	0.961(0.942∼0.981)	<0.001	0.958(0.938∼0.978)	<0.001
LDH	0.513(0.400∼0.657)	<0.001	0.475(0.372∼0.605)	<0.001
NC (HNC vs. LNC)	1.828(1.409∼2.371)	<0.001	-	-
NP(HNP vs. LNP)	-	-	1.475(1.106∼1.967)	0.008

Abbreviation: NC, neutrophil counts; NP, neutrophil percentages; HR: hazard ratio; CI: confidence interval; LNC: low NC (pretreatment NC<=8); HNC, high NC (pretreatment NC>8); LNP, low NP (pretreatment NP<=0.85); HNP, high NP (pretreatment NP>0.85); BSC, best supportive care; PCR, palliative chemoradiotherapy; ECOG, Eastern Cooperative Oncology Group.

* The potential prognostic factors used for adjustment in Cox regression model include: Age; Gender; Family history; Nutrient status; ECOG; Primary tumor site; Tumor stage; Comorbidity, Palliative care; Albumin; LDH.

**Figure 3:**
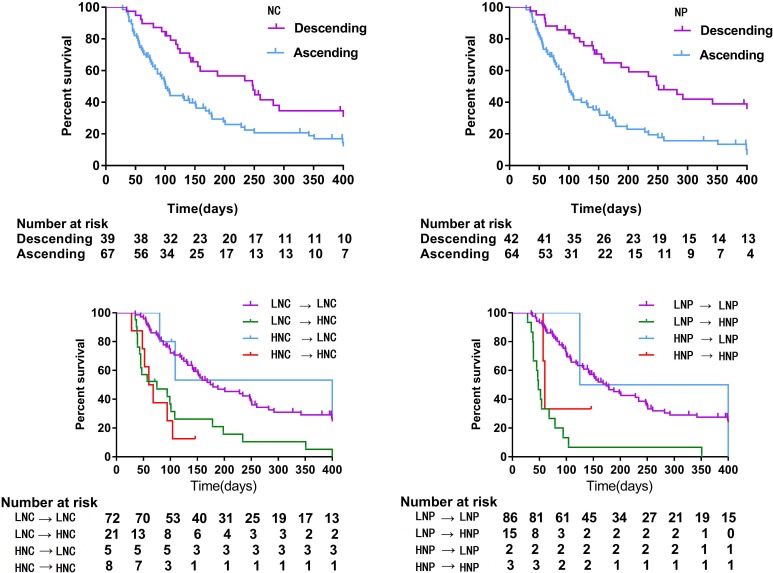
Overall survival of patients under palliative care stratified by changes in NC and NP (cohort 2) Abbreviation: NC, absolute neutrophil count; NP, percentage of neutrophils; LNC, low NC (pretreatment NC < = 8); HNC, high NC (pretreatment NC > 8); LNP, low NP (pretreatment NP < = 0.85); HNP, high NP (pretreatment NP > 0.85).

To validate the prognostic significance of the dynamic changes in NC and NP, we studied cohort 2, in which patients were divided into ascending and a descending groups based on changes in the NC or NP. Multivariate analyses showed that an increased NC or NP was significantly associated with a poor OS (*HR*: 1.837, 95% CI: 1.096∼3.079, and *HR*: 3.268, 95% CI: 1.848∼5.778, respectively) (Table 4). Patients with a descending NC or NP had a significantly longer survival time than those with an ascending NC or NP (248 days (155-409) *vs*. 101 days (83-151), *P* < 0.001; and 251 days (159-539) *vs*. 99 days (83-132), *P* < 0.001) (Figure 3). We hypothesized that patients with a pretreatment LNC or LNP that transitioned into an HNC or HNP after a second admission to the hospital would have the worst OS. Further subgroup analyses were performed on the associations between changes in NC or NP and OS. When demographic and disease-specific factors were adjusted, significant results were obtained (Table 4). The hazard ratios of patients with a pretreatment LNC or LNP that became an HNC or HNP after palliative care were approximately 2.77 and 7.54, respectively. The Kaplan-Meier analyses were consistent with those results (Figure 3).

**Table 4:** Adjusted HRs for overall survival stratified by changes in NC and NP in cohort 2 (*N* = 106)

NC	NC Model*		NP	NP Model*	
	*HR* (95% CI)	*P* value		*HR* (95% CI)	*P* value
Descending	Reference		Descending	Reference	
Ascending	1.837(1.096∼3.079)	0.021	Ascending	3.268(1.848∼5.778)	<0.001
LNC→LNC	Reference		LNP→LNP	Reference	
LNC→HNC	2.772(1.560∼4.928)	0.001	LNP→HNP	7.536(3.610∼15.73)	<0.001
HNC→LNC	0.429(0.110∼1.673)	0.223	HNP→LNP	0.421(0.087∼2.038)	0.282
HNC→HNC	2.537(1.030∼6.249)	0.043	HNP→HNP	2.497(0.545∼11.44)	0.239

Abbreviation: NC, neutrophil counts; NP, neutrophil percentages; HR: hazard ratio; CI: confidence interval; LNC: low NC (pretreatment NC<=8); HNC, high NC (pretreatment NC>8); LNP, low NP (pretreatment NP<=0.85); HNP, high NP (pretreatment NP>0.85).

* The potential prognostic factors used for adjustment in Cox regression model include: Age; Gender; Family history; Nutrient status; ECOG; Primary tumor site; Tumor stage; Comorbidity, Palliative care; Albumin; LDH.

## DISCUSSION

To our knowledge, this is the first study that reports the relationship between neutrophils and outcomes of patients with advanced cancer who are under palliative care. The principal findings of our study are as follows: a) a higher pretreatment NC or NP is a strong independent predictor of an adverse outcome in patients with advanced cancer; b) patients with an increased NC or NP after palliative care also show a poorer OS during the follow-up period. These results suggest that peripheral blood neutrophils may serve as a novel risk stratification surrogate marker in patients with advanced cancer who are under palliative care.

Despite the great steps that have been achieved, the treatment of patients with advanced cancer is still associated with a high probability of failure. Survival estimations are urgently needed for risk stratification and further clinical decision-making. However, until now, no prominent or adequate biomarkers have been widely used. Peripheral neutrophils, which are a novel marker of inflammation, are routinely measured in almost all hospitals. The peripheral NC has been reported to predict poor clinical outcome in various cancers, including gastric cancer, nasopharyngeal carcinoma, urinary tract carcinoma, lung cancer and ovarian cancer [6, 8–10]. However, scarce evidence was found between the percentage of neutrophils and survival of cancers [11, 12]. Controversial issues were also found between neutrophil count or percentage with biochemical recurrence and disease free survival in prostate cancer patients [13]. In addition, neither the count nor the percentage parameters of peripheral neutrophils has been investigated in a palliative care setting for the prognosis of patients with advanced cancer patients.

In the present study, we evaluated the prognostic impact of neutrophil count and percentage in an institution with a clinical practice of palliative care for patients with advanced cancer. We first established cut-off values for neutrophil count and percentage as prognostic tools to predict the outcomes of advanced cancer patients, respectively. As shown, a NC equals 8.0×109/L was defined as the cut-off point. This value is consistent with that in previous clinical studies that investigated the significance of peripheral NC in human cancers, in which cut-off values of 3.9 ∼ 8.0×109/L were used [8, 9]. In addition, the cut-off value for NP was set at 85.0%, which is similar to the value in a previous report (82.1%) [16]. Our results showed that both initial neutrophil count and percentage were significantly correlated with the ECOG score, which suggests that patients with an initial high neutrophil count and percentage were more likely to be in a poorer physical state, and therefore, have a poorer prognosis. Moreover, patients with a high NC or a high NP had significantly shorter lifespan than their peers. The survival curves were almost identical for NC and NP. In agreement with these findings, according to the OS analysis, both the high neutrophil count and the percentage displayed consistently higher HRs than low neutrophil parameters in patients with advanced cancer (*P* < 0.001), which suggests patients with high neutrophils had a poorer survival than those with low neutrophils. In addition, gender, tumor stage, albumin, LDH, and ECOG score are also found to be risk factors for death in patients with advanced cancer, which is consistent with data from previous studies [17–20]. Collectively, these results indicate that a high pretreatment neutrophil count or percentage is a strong and independent predictor of poorer outcomes among patients with advanced cancer in a palliative care setting. Furthermore, pretreatment NC was equally predictive of prognosis as the NP in patients with advanced cancer when each was analyzed separately.

No research has examined the dynamic changes in neutrophils for advanced cancer patients in a palliative care setting. Accordingly, this study was performed to address these gaps in knowledge using the strengths of dynamic change. Our study extends the aforementioned findings and shows that the patients with an ascending neutrophil count or percentage had a significantly decreased OS compared with those with a descending neutrophil count or percentage, which further emphasizes the impact of neutrophils on patient outcomes. Additionally, our results revealed that patients with advanced cancer in a palliative care setting who transitioned from a low NC or NP to a high NC or NP were at a greater risk for poor outcomes. Taken together, as convenient and intuitive markers, pretreatment and dynamic peripheral neutrophils demonstrated the ability to predict aggressive clinical outcomes in patients with advanced cancer. The results of the present study may reveal prognostic variability of the pretreatment level of neutrophils and the dynamic changes in peripheral neutrophils, irrespective of the format used, because it is clear that the NC, accounts for the majority of the leukocyte count.

The relationship between human cancer and neutrophils has stimulated increased research interest in a wide range of topics. As we know, cancer-related inflammation has been identified as the seventh hallmark of tumor development [4]. Substantial evidence suggests that the presence of inflammatory cells plays a critical role in human tumors [21, 22], and that neutrophils are emerging as central players in the inflammatory tumor microenvironment [23]. The pro-tumor effects of neutrophils are mediated by different mechanisms [24]. First, cancer cells release many myeloid growth factors or chemokines that result in the production of neutrophils [25]. Normally, only fully differentiated neutrophils are recruited from the bone marrow into the peripheral circulation, whereas immature neutrophils are released into the peripheral blood under inflammatory conditions [26]. Second, circulating neutrophils have been shown to secrete various cytokines, including matrix metalloproteinases, which causes vascular endothelial growth factor to be released from the extracellular matrix, which in turn promotes angiogenesis [27, 28]. Neutrophils have also been shown to secret interleukins, which induce a chronic inflammatory state, and arginase 1, which inhibits CD8 T cells and creates an immunosuppressive state [29, 30]. Reactive oxygen species released by neutrophils can damage DNA, which induces genotoxic effects in tumor cells [31]. Further, neutrophils can secrete enzymes that degrade the basement membrane and promote tumor cell invasion through the basement membrane [32]. They also secrete enzymes that promote the survival of tumor cells *via* the induction of tumor cell aggregation and adherence to arrested neutrophils, which then promotes extravasation of the tumor cells [33]. Together, these factors contribute to tumor-related angiogenesis, tumor proliferation, migration, invasion, and metastasis. As markers of inflammation, neutrophils indicate increased tumor burden and aggressive tumor biology, and thus reflect the prognosis.

Although this study provides important insights into the association between peripheral neutrophils and the outcome of patients with advanced cancer, some limitations of this study need to be addressed. First, this study is limited by its retrospective, single-center design, which carries several inherent limitations including selection bias, possible confounding factors, and relatively low sample size. Second, additional inflammatory biomarkers (e.g., erythrocyte sedimentation rate, C-reactive protein, and interleukins) were not evaluated as part of the multivariable analysis since they are not routinely measured in our clinical practice. Thus, larger prospective multi-institutional studies that include more clinical markers are needed to validate the associations between peripheral neutrophils and poor prognosis.

In conclusion, we demonstrated for the first time that peripheral neutrophils are a strong and independent predictor of shorter survival for patients with advanced cancer following palliative care. As a readily available and inexpensive inflammatory marker, peripheral neutrophils merit further investigation in future clinical practice for risk stratification and may allow more proper clinical decision-making of advanced cancer patients.

## MATERIALS AND METHODS

### Data collection and study cohort

Consecutive patients treated at the palliative care unit of Fudan University Shanghai Cancer Center (FUSCC), Shanghai, China between July 2013 and October 2015 were retrospectively reviewed. Demographics (age, gender), medical history (comorbidities, smoking status and family history), tumor-related factors (primary tumor site and tumor stage), nutritional and physical status (Eastern Cooperative Oncology Group, ECOG score) were obtained from their medical records of the patients. White blood cell count and its differential counts were performed 1-3 days before the start of palliative care. The NP was calculated as the absolute NC divided by the total WBC count. An unintentional weight loss > 5% in the previous 3 months or a food intake below 75% of the normal requirement in the preceding week were considered to be an abnormal nutritional status according to the ESPEN guidelines for nutrition screening [34]. The presence of comorbidity was defined as self-reported cardiac disease, hypertension, diabetes, or any cerebrovascular disease, that might affect or involve systemic inflammation.

The methodology and criteria that were used to identify the two retrospective cohorts have been previously reported [14]. Briefly, advanced cancer patients with complete medical records during the study period were categorized into cohort 1. In this cohort, we examined the associations of several potential risk factors with overall survival (OS). Patients with a readmission data were categorized into cohort 2, and the last follow-up date was in December 2015. The effects of changes in NC and NP on OS were evaluated. This study was approved by the Ethics Committee of FUSCC. Informed consent was waived because of the retrospective nature of the study.

### Statistics analysis

Data are presented as the mean ± standard deviation (SD) for continuous variables and as totals and frequencies for categorical variables. The distribution of the clinicopathological features was tested using the Wilcoxon sum rank test, chi-squared or Fisher's exact test as appropriate. A logarithmic transformation was applied to pretreatment NC because of its skewed distribution (*P* < 0.001). The Kaplan-Meier method was used to plot the survival curves, and a Log-rank test was conducted to test differences between groups. Cox proportional hazards models were employed to estimate the magnitude of the association between the clinicopathological features and OS. X-tile version 3.6.1 (Yale University, New Haven, CT, USA) was used to determine optimal cut-off values. More precisely, the X-tile divided subjects into two subgroups on every possible cutoff point, and then, a Log rank test was used to calculate a chi-square statistic. The optimal cutoff point was selected based on a minimum *P* value with the maximum chi-square statistic. The concordance index (C-index) [35], which ranges from 0 to 1.0, was used to assess the discriminative ability of the cut-off value. All tests were two-sided and *P* values less than 0.05 were considered statistically significant. Statistical analyses were performed using SAS 9.4 (Cary, NC, USA) and R software version 3.3.1 (Institute for Statistics and Mathematics, Vienna, Austria).
